# Emergency burr holes: "How to do it"

**DOI:** 10.1186/1757-7241-20-24

**Published:** 2012-04-02

**Authors:** Mark H Wilson, David Wise, Gareth Davies, David Lockey

**Affiliations:** 1 London's Air Ambulance, The Helipad, The Royal London Hospital, London E1 1BB, UK; 2 Emergency Department, Barts and the London NHS Trust, London E1 1BB, UK; 3 Department of Neurosurgery, Imperial Hospitals NHS Trust, London W2 1NY, UK; 4 School of Clinical Sciences, Bristol University, Bristol BS10 5NB, UK

**Keywords:** Burr Hole, Extradural haematoma, Remote Medicine

## Abstract

This paper describes a simple approach to emergency burr hole evacuation of extra-axial intracranial haematoma that can be used in the uncommon situation when life saving specialist neurosurgical intervention is not available.

## 

No patients involved

This paper describes a simple approach to emergency burr hole evacuation of extra-axial intracranial haematoma that can be used in the uncommon situation when life saving specialist neurosurgical intervention is not available.

Rapidly expanding intracranial haematomas associated with fixed dilated pupils are rapidly fatal. A recently fixed dilated pupil with corresponding imaging evidence of an extra-axial haematoma is considered an indication for emergency targeted burr hole placement.

Extra-axial haematomas (extradural/subdural) by definition are outside the brain and hence are not a primary brain injury. It is the delay in removing the compression of the brain by the clot that causes brain injury and death.

Ideal treatment is provided by immediate specialist neurosurgical care. However in many parts of the world, this is not always available and the risks of delay associated with secondary transfer have to be balanced with the risks of the procedure being done by a non-specialist. At one UK neurosurgical centre, the median transfer time was 5.25 hours for patients with extradural haematoma and 6 hours for subdural haematoma [1]. The prolonged transfer of a patient with fixed/dilated pupils is unlikely to have a good outcome. Transfer of this type of patient is analogous to transferring a patient with other time critical but reversible pathology such as a tension pneumothorax. There are many reports of non-specialists successfully performing emergency burr holes [2]. These are often done with household drills and other makeshift tools which, when successful, has created media interest [3]. Although there have been significant technical advances in the safety of the procedure since the time of "exploratory" burr holes, there has simultaneously been a reduction in the number of surgeons either having experience in or being willing to perform the procedure. A number of general surgeons working in remote areas of Australia are more confident in performing simple neurosurgical procedures even though they may have no greater training than general surgeons closer to neurosurgical centres [4]. This may paradoxically result in more optimal management in more remote regions. With adequate training and skill retention, burr hole drainage of acute extradural haematomas can be performed by non-neurosurgeons [5]. Despite this, We must stress that this procedure should only be performed if it is not possible to transfer a patient to a more appropriate centre in a timely manner and that this procedure must not delay transfer. A previous study has demonstrated that attempts by local, non-trained personnel can result in delay and worse outcome [6]. This must not occur.

The authors describe a simple approach to burr hole placement. The important considerations here are that the burr hole should be targeted (not exploratory), should be done using the correct tools (and in particular a perforator drill bit with a clutch mechanism, Figure 1) and should not unduly delay the transfer of the patient who will usually still require an urgent craniotomy.

**Figure 1:**
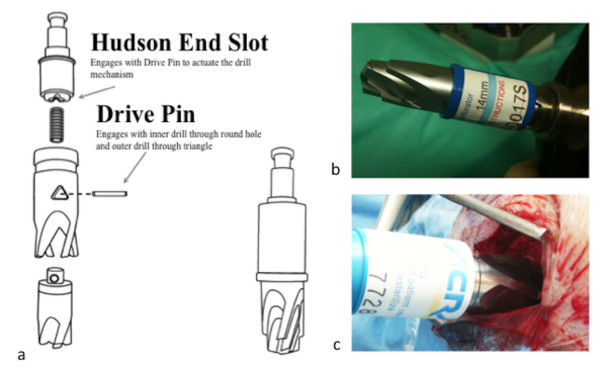
**a: A technical drawing of the clutch mechanism of a "perforator" drill bit (courtesy of Codman, Johnson and Johnson). b**: photograph of "perforator" clutch drill bit. **c**: burr Hole being performed.

## Emergency "Burr Hole" Craniostomy

### Indication

Patient with reduced GCS (< 8) with imaging evidence of an extra-dural haematoma causing midline shift and unequal pupils when timely neurosurgical intervention is not possible. Attempts should always be made to discuss the images and necessity for a procedure with a neurosurgeon.

### Contraindications

• GCS > 8

• No Imaging*

• Neurosurgical intervention available in a reasonable time frame.

* Very high clinical suspicion (e.g. a palpable fracture with an ipsilateral fixed pupil), in an area remote from CT imaging may be an exception to this. In the future devices such as the Infrascanner™ (a handheld portable device designed to detect extra-axial haematoma using near infra-red light) may lessen the need for formal CT imaging in the emergency setting. Currently however, a CT scan should always be performed, especially if a non-neurosurgeon is contemplating performing the procedure.

### Equipment

The surgical equipment is standard (knife, self retainer, swab, drill, sharp and blunt hock and second knife). If possible, bipolar diathermy should be set up. The hand held drill (which can be a Hudson-Brace or air powered drill) should have a specific perforator (e.g. a 14 mm perforator clutch drill bit (26-1221, Codman, Johnson and Johnson, Chicago, USA), Figure 1. All equipment should be stored together ready for use in an emergency department or operating theatre. Importantly, in preparation, check the drill bit fits the drill.

### Procedure

*Note: It may be useful to nominate a person to read out the components of this guide as the procedure is being performed*

**Figure 2:**
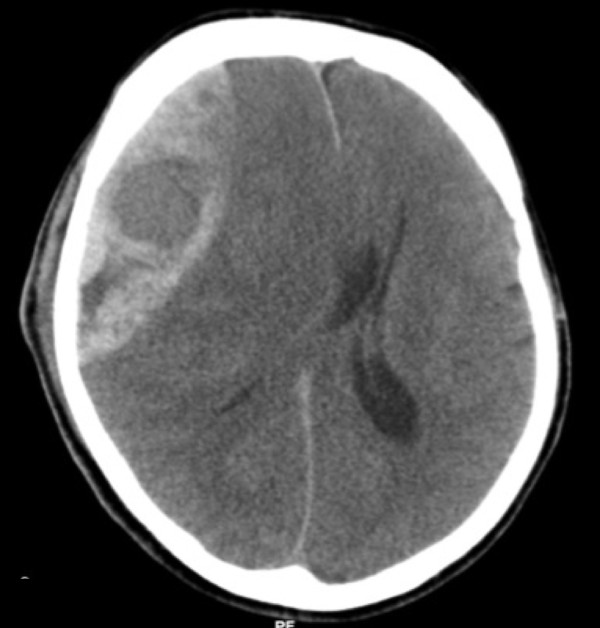
**CT scan demonstrating an extradural haematoma**.

**Figure 3:**
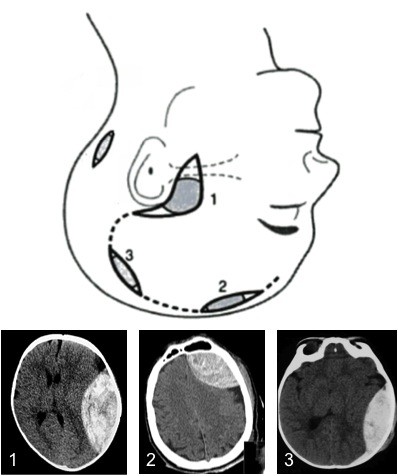
**Diagram demonstrating position of standard burr holes (1, temporal (above zygoma), 2 frontal (over the coronal suture, approx 10 cm behind and in the mid-pupillary line) and 3 parietal (over the parietal eminence)**. CT Images correspond. A posterior fossa burr hole can be used in the extremely rare cases of posterior fossa extradural haematoma.). See text for indications, requirement for imaging and requirement for neurosurgical discussion. (Image adapted from Head Injuries p134, Mark Wilson Oxford Desk Reference of Trauma Ed Smith, Greaves and Porter 2011).

1) Ensure indications are appropriate

2) Ensure patient is supine and physiologically optimised (intubated, ETCO2 4.5kPa, normotensive, c-spine protection, mannitol/hypertonic saline as directed by neurosurgeon).

3) Confirm position of haematoma on CT scan and be able to view images while performing procedure (Figure 2). Mark the patients shoulder that corresponds to the side of the haematoma. Haematomas most commonly occur in the temporal region. Frontal, parietal, and rarely posterior fossa haematomas also occur. Figure 3 demonstrates the standard position of burr holes for each of these situations. These positions can be modified slightly in light of the scan. It is important that the burr hole is over the centre of the haematoma. Count down the number of slices from the top (and multiple by slice thickness) to the centre of the haematoma to calculate how many centimetres below the vertex the burr hole should be.

4) Shave a strip of approximately 5 cm of hair where the burr hole is to be made.

5) Mark a 3 cm line of incision.

6) Clean the area with betadine/chlorhexidine

7) Make an incision straight down to bone. Bleeding (e.g. from the superficial temporal artery) can be controlled with direct pressure while continuing the procedure.

8) Push the periosteum off the bone with knife/swab

9) Insert self-retaining retractor

10) Push down firmly with drill and start drilling keeping drill perpendicular to the skull. Ensure an assistant is holding the head still and ideally apply saline wash as you drill.

11) Keep going - do NOT stop (as this will disengage the clutch mechanism which can be difficult to re-engage manually)

12) Drill until the drill bit stops spinning. Remove drill.

13) Use blunt hook to remove remaining bone fragments.

14) Extradural blood should now escape.

15) If the blood is subdural, very carefully open the dura using a sharp hook to tent the dura up, and a new sharp knife to incise the dura in a cruciate manner. Subdural blood is likely to be more clotted and difficult to extrude than extradural. Manual removal of clot (e.g. with forceps or very careful suction) could be considered, but may damage brain and is unlikely to remove sufficient haematoma. If no blood is found either extra or sub-durally, stop, check side, and check location of hole. DO NOT DELAY TRANSFER.

16) If fresh blood is continuing to ooze from the wound, do NOT try to tamponade. Leaving the self-retainer in place may stop the bleeding. Try to diathermy skin edges; if not available, apply direct pressure to wound edges during transfer.

## Discussion

The cranial procedure of burr hole placement has become the sole domain of neurosurgeons particularly as they can deal with surgical complications. As such, non-neurosurgeons are no longer familiar with the technique. This creates a therapeutic vacuum for patients remote from specialist care who meet the criteria for urgent burr hole drainage.

Central to the ability of non-neurosurgeons to successfully performing burr holes is the development of *clutch *drill-bits (Figure 1). These cause the drill to disengage on penetrating the inner table of the skull so that the risk of "plunging" is minimised making the procedure considerably safer. If a haematoma is not relieved, the patient should not have come to any additional harm *providing transfer to a neurosurgical centre is not significantly delayed*. In remote areas of Australia, when such neurosurgical procedures are performed by non-neurosurgeons, outcomes are acceptable [7]. Even in less remote situations non-neurosurgeons in district general hospitals in the UK have historically carried out emergency craniotomy [8].

For many years it has been known that earlier surgical intervention is of benefit in the management of head trauma when an extra-axial collection can be removed [9]. In the future, near infra-red/ultrasound devices or mobile CT, may mean that extra-axial collections can be detected in remote locations. This will not be of benefit unless the time to surgical relief of increased Intracranial Pressure is also shortened.

While attempting to remove the mystique and anxiety surrounding emergency burr hole placement, we emphasise the importance of avoiding inappropriate intervention. However, when faced with a situation where mortality approaches 100%, a simple technique, using the correct equipment can be robust, safe and life-saving even in the hands of non-specialists.

## Competing interests

The authors declare that they have no competing interests.
